# A custom-made vitreoretinal surgical simulator using a silicone mold

**DOI:** 10.1186/s12886-023-03070-5

**Published:** 2023-07-11

**Authors:** Takashi Nagamoto, Hirohisa Kubono, Mari Kawamura, Kotaro Suzuki

**Affiliations:** 1 Department of Ophthalmology, Keiyu Hospital, 3-7-3 Minatomirai, Nishi-ku, Yokohama-city, 220-8521 Kanagawa Japan; 2 Department of Ophthalmology, Keio University School of Medicine, Tokyo, Japan

**Keywords:** COVID-19, Dry lab, Ophthalmology residents, Surgical simulator, Trainees, Virtual reality

## Abstract

**Purpose:**

We constructed a custom-made vitreoretinal surgical simulator using a silicone mold and described its practicality.

**Methods:**

We obtained spherical silicone molds, mannequins, and spray material from an internet-based vendor and combined them with expired surgical instruments to complete the simulator. Vitreoretinal experts confirmed the practicality of the simulator after simulated vitrectomy, and the results of the questionnaires were confirmed by nonvitreoretinal experts.

**Results:**

Vitreoretinal experts observed that the simulated eyeball and the actual eyeball were similar in size and rigidity and that the intraocular practice swing seemed to be useful for the prevention of complications. The semitransparency and open-sky structure of the silicone material ensured visibility. The simulated membrane, which was spray glue, provided an excellent peeling sensation. In the results of the nonvitreoretinal experts’ questionnaires, the average scores of all items were generally high, which supported the claims of the simulator’s usefulness.

**Conclusion:**

This report describes the simplicity and cost-effectiveness of our custom-made simulator and its contribution in creating an ideal training environment that does not necessitate travel to special facilities that offer a large number of pig eyes and vitreous surgical machines. The simple shape seems to allow many possibilities, and further verification at multiple facilities is necessary.

**Supplementary Information:**

The online version contains supplementary material available at 10.1186/s12886-023-03070-5.

## Introduction

To improve ophthalmic surgeons’ technique, it would be desirable to provide surgical training that is remarkably simple and affordable and can be casually performed every day. Wet lab training, involving the use of pig eyes, is a common method of practicing cataract surgery [1]. Vitrectomy using pig eyes is also possible [2], but there is no consensus on vitreoretinal surgery training in Japan [3]. It is speculated that there are many countries with similar circumstances. Furthermore, the COVID-19 pandemic has restricted the use of numerous wet lab facilities worldwide [4]. Therefore, such a complex situation may cause complications to occur because of unskilled surgeons.

Practical methods for vitreoretinal surgery, other than wet lab procedures, have been previously reported [3, 5–12]. Virtual reality (VR) machines that support the visualization of various training surgical techniques are attractive simulators [5–8]. Other surgical simulators that enable the use of actual surgical instruments are also useful [3, 9–12]. However, young surgeons have requested that more easy-to-use eye model simulations be created, and we have therefore explored novel surgical simulators.

The purpose of this report is to describe the practicality of a simple custom-made vitreoretinal surgical simulator that is cost-effective and easily obtained.

## Materials and methods

**Fig. 1:**
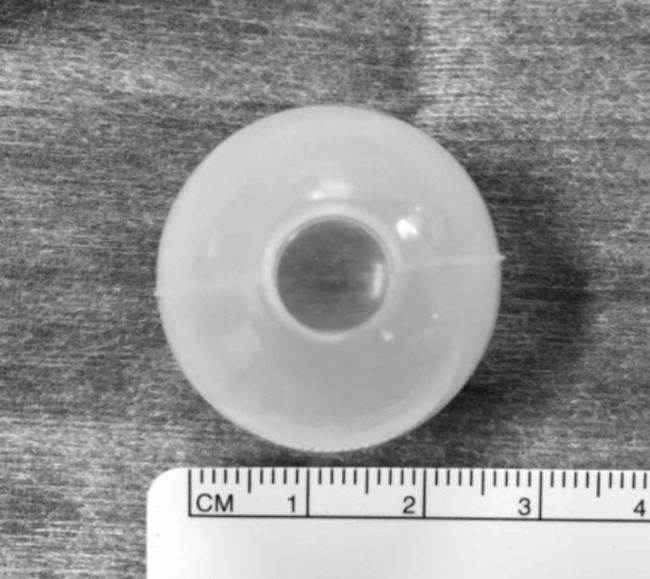
Spherical silicone mold. We used a spherical silicone mold that had an 8-mm hole at the top and a semitransparent spherical form with a 25 mm inner diameter and a 1 mm thickness

**Fig. 2:**
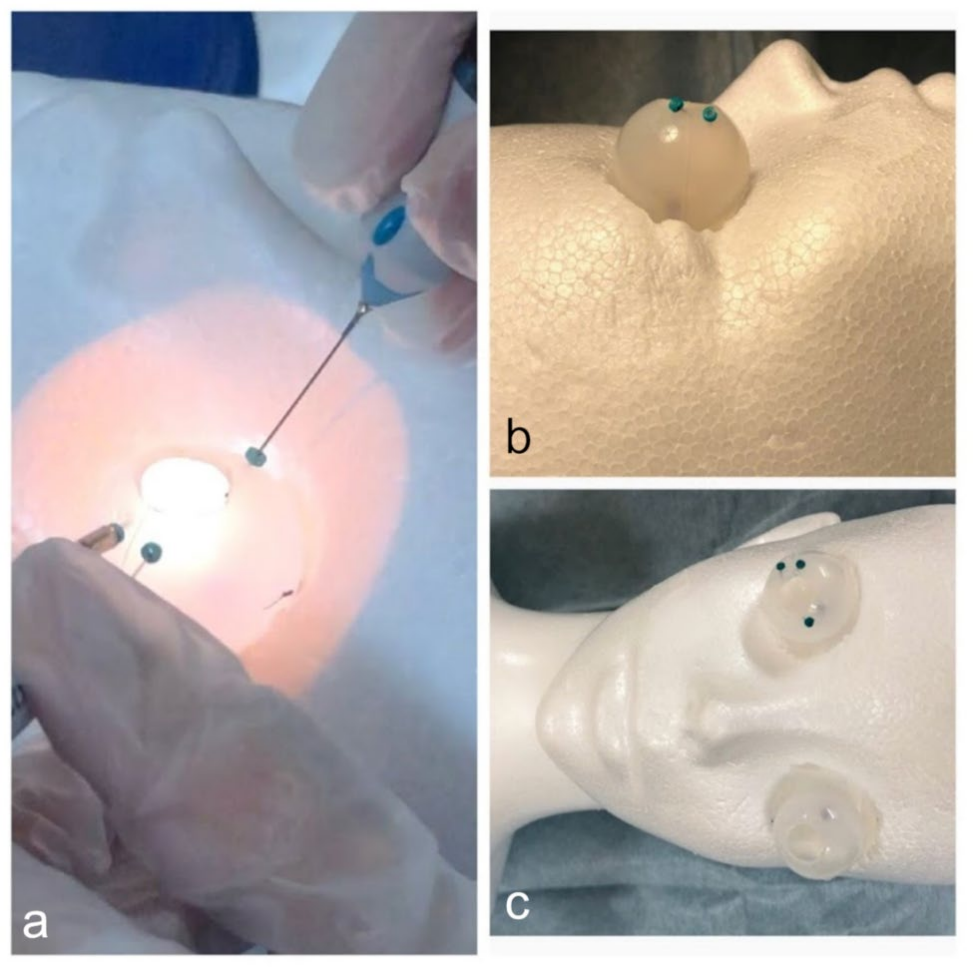
Overviews of our simulator. **a** Photo taken while using the simulator. **b** Side view. **c** Front view

One of the most important structures of this simulator is its spherical silicone mold, which makes it an ocular substitute. It is generally used as a tool to make accessories by pouring resin into the mold. The material we used had an 8 mm hole on the top and a semitransparent spherical form, with a 25 mm inner diameter and a 1 mm thickness (Fig. 1). The silicone mold is a flexible material that can be easily cut with scissors; we enlarged the hole by several mm and brought it close to the corneal diameter. Furthermore, after pinning the spherical silicone mold onto the optic nerve of the Styrofoam mannequin head, whose orbit was already dug, a very precise human eye model was constructed (Fig. 2).

The intraocular membrane was mimicked by spray material (Scotch Spray Glue 55, 3 M Japan Limited, Shinagawa-ku, Tokyo, Japan). The spherical silicone mold was reversed and lightly sprayed on the bottom. Approximately 10–20 min for drying enabled us to use it as our very thin material.

We added sterilized or expired disposable vitreous surgical instruments that were used in the facility to this substitute eyeball. In our hospital, we used vitreous forceps (25G Grieshaber Advanced DSP Tips ILM Forceps, Alcon Laboratories Inc., Fort Worth, TX, USA), cutter and light guide probes (25G Constellation Vision System, Alcon Laboratories Inc., Fort Worth, TX, USA), and other devices.

For observation, we used a surgical microscope (OPMI Lumera 700, Carl Zeiss Meditec, Oberkochen, Germany) and a wide viewing system (Resight, Carl Zeiss Meditec, Oberkochen, Germany).

The practicality was investigated as follows: (1) vitreoretinal experts reviewed the usability of this simulator in the same way as for regular surgery procedures, and (2) nonvitreoretinal experts who used this simulator for practice evaluated the simulator by completing a questionnaire regarding the surgical training (Table 1). Parts I and II asked questions about the preparation, and Parts III-XI asked questions about the actual procedures. With respect to the questions in Part XII, we used these questions to investigate whether they were suitable for continuous practice. Primary surgeons who performed a minimum of 30 vitrectomies for rhegmatogenous retinal detachment and 30 scleral bucklings and who subsequently had continuous surgical practice as vitreoretinal consultants were classified as vitreoretinal experts [13].

**Table 1:** Questionnaire for nonvitreoretinal experts

Contents of questions
I. Is the cost for the preparation appropriate?
II. Are the time and effort required for the preparation appropriate?
III. Did you understand the maneuver of creating the port?
IV. Did you understand the sense of distance between the cutter and the retina during a vitrectomy?
V. Did you understand how to illuminate the surgical field using the light guide during a vitrectomy?
VI. Did you understand the coordinated movement of both hands during a vitrectomy?
VII. Did you understand the operation that does not unknowingly distort the eyeball during a vitrectomy?
VIII. Did you understand how to handle the instrument in the maneuver of indentation?
IX. Did you understand how to handle the instrument during membrane peeling?
X. Did you understand how to handle the instrument when closing the wound?
XI. Was it easy to understand the model procedure for senior doctors?
XII. Is the model suitable for continuous vitreous surgery practice?

Surgeons who did not continue surgical practice as vitreoretinal consultants and did not perform at least 30 scleral bucklings and 30 vitrectomies for rhegmatogenous retinal detachment were classified as nonvitreoretinal experts.

These items were evaluated as follows. 1: Extremely bad, 2: Bad, 3: Neither, 4: Good, 5: Extremely good.

## Results

Four surgeons were classified as vitreoretinal experts, and five surgeons were classified as nonvitreoretinal experts in our hospital. Reviews of this surgical simulator, using silicone molds, were summarized as follows for each procedure (Online Resource 1 has instructions provided by H.K., a vitreoretinal expert, on how to use this simulator).

## *Reviews by vitreoretinal experts*

**Fig. 3:**
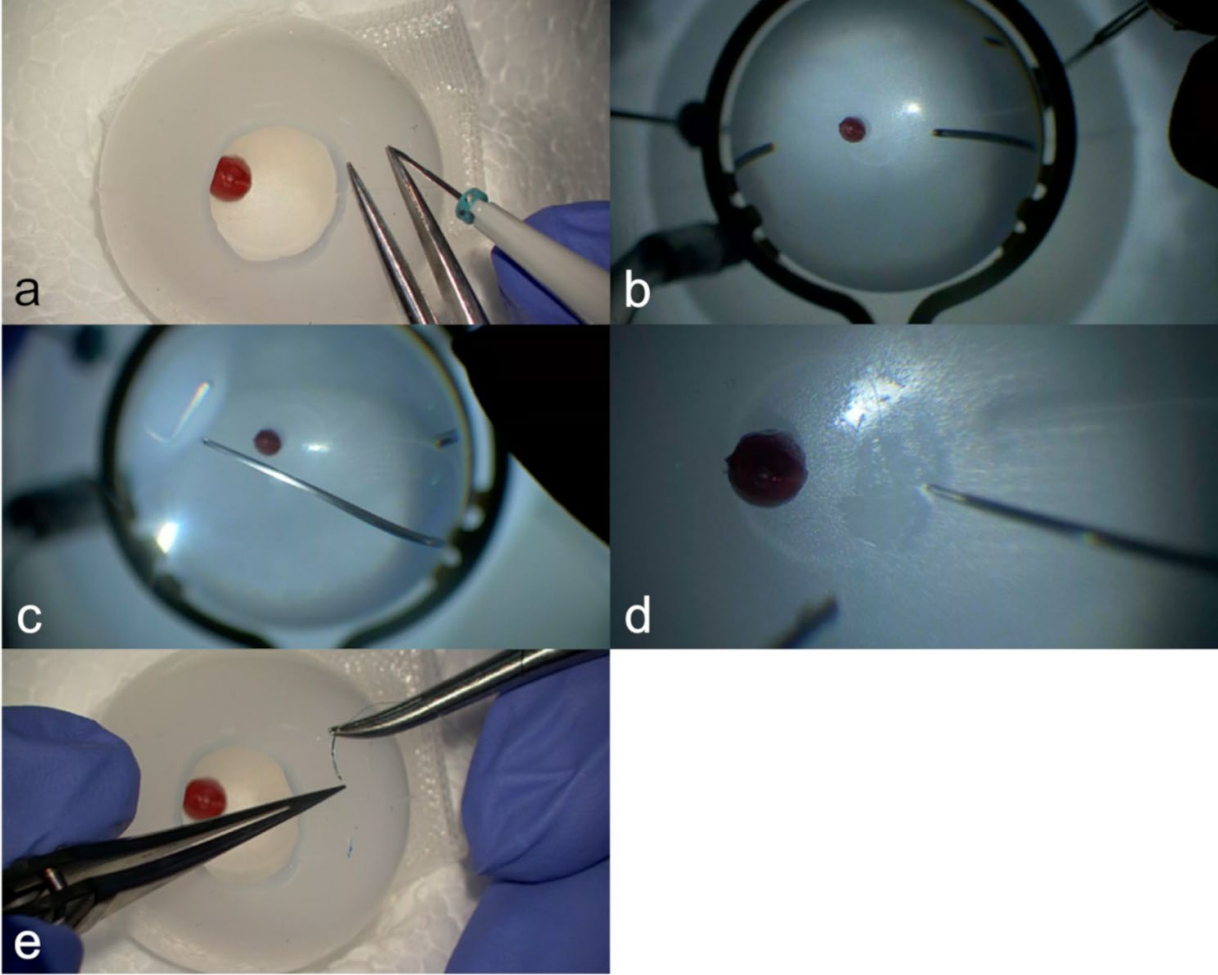
Simulated vitreoretinal surgery using our simulator. **a** Sclerotomies. **b** Vitrectomy. **c** Vitreous base shaving under indentation. **d** Membrane peeling. **e** Wound closure

The experts made 3 ports 3–4 mm from the simulated corneal limbs (Fig. 3a). Because they considered the rigidity of this material to closely resemble that of a real eyeball, this practice may mimic the sensation of performing sclerotomies, such as the angled incision technique.

Core vitrectomy, posterior vitreous detachment induction and peripheral vitrectomy were performed while taking care not to unknowingly move or distort the simulated eyeball (Fig. 3b). Its open-sky structure made the experts feel that the visibility was excellent. They felt that they had a better surgical field, which was achieved with the help of the wide viewing system. However, with only a surgical microscope, even with the naked eye, they could obtain enough visibility. There were some differences between this simulator and an actual eyeball due to the lack of refractivity of the anterior segment, but it was sufficient and useful for practice. This stroke training seems to facilitate the actual approach from all directions into the vitreous cavity while maintaining a good surgical field, which is important for the prevention of intraoperative complications.

The experts moved a cutter probe and indented each quadrant of the spherical silicone mold using a chandelier lighting system (25G Vivid Chandelier, Synergetics, Inc., St, Charles, Missouri, USA) (Fig. 3c). The material, which features semitransparency and moderate rigidity, was expected to help inexperienced surgeons gain a sense of the coordinated movement of a cutter probe and a scleral depressor.

The membrane made of Scotch Spray Glue 55 was peeled off by vitreous forceps (Fig. 3d). The experts felt that its touch resembled that of the sticky epiretinal membrane (ERM). This feeling induced excitement and allowed trainees who had not experienced vitreous surgery to “peel” the membrane.

The experts removed the cannulas and closed the wounds with surplus surgical sutures (Fig. 3e). This practice contributed to the acquisition of skills to create smooth sutures, although the frictional resistance was slightly increased compared with that of actual eyeballs.

Although Online Resource 1 did not demonstrate this, if a lens (HHV DISPO, Hoya, Shinjuku-ku, Tokyo, Japan) was fitted into the simulated cornea to create a closed space and a gel-like substance was poured, then a cutter probe and infusion system was driven. This imitated vitreous body resection, using a foot pedal, allowed the experts to achieve a more realistic sensation, but the disposable pack was impractical due to its cost and effort.

## *Evaluations by nonvitreoretinal experts*

The results of the questionnaire are shown in Table 2. The mean scores of all the items were 3 or higher, which was a high value. The evaluations of Parts III-XI, regarding the actual procedure, were widely varied, but the evaluations of Parts I and II, regarding preparation, and part XII, regarding continuity, were less varied.

**Table 2:** Results of the questionnaire completed by nonvitreoretinal experts

Question number	I	II	III	IV	V	VI	VII	VIII	IX	X	XI	XII
**Mean ± SD**	4.8 ± 0.4	4.4 ± 0.5	4.8 ± 0.4	3.8 ± 1.0	3.4 ± 1.4	4.0 ± 1.1	3.6 ± 1.4	3.6 ± 0.8	3.8 ± 0.7	4.2 ± 0.7	4.2 ± 1.2	4.6 ± 0.8
**Range**	4 to 5	4 to 5	4 to 5	2 to 5	1 to 5	2 to 5	1 to 5	2 to 4	3 to 5	3 to 5	2 to 5	3 to 5

5 surgeons were classified as nonvitreoretinal experts in our hospital. Abbreviation: *SD*, standard deviation.

## Discussion

On occasion, inexperienced trainees have to perform surgery on patients before their skills are well developed [14]. Our simple simulator, using a spherical silicone mold, may be a solution for such a problem in vitreoretinal surgery. This reusable silicone material has moderate flexibility and similarities to the ocular axial length [15] and the thickness of the sclera [16]. Moreover, its semitransparency and open-sky structure are suitable for trainees to observe the model procedures of senior doctors as well as their own procedures. The results of the questionnaire were generally good, seemed to support the abovementioned facts and proved that the simulator corresponded to various vitreous surgery procedures.

Referring to past reports [9, 10], we reproduced extremely thin membranes by spray material, Scotch Spray Glue 55. Moderate spraying gave vitreoretinal experts a sticky ERM-like sensation, which seemed to be easily reproduced and of sufficient quality and helped nonvitreoretinal experts to understand the procedure. We tried numerous kinds of tape and paint materials, but the silicone material prevented them from adhering well. Other unknown ingredients with the potential to produce an excellent membrane have not been sufficiently examined; thus, further studies on membrane materials are necessary.

Traditionally, surgical practice with pig eyes has been common [1, 2], but there are problems in terms of preservation, hygiene, and reusability. Recently, the progress of vitreoretinal surgical training appliances using VR, for example, EyeSi as a representative, has been remarkable [5–8]. However, EyeSi is extremely expensive and not generally used yet [17]. Dry lab surgical simulators, based on previous reports [3, 9–12], have been fascinating for understanding direct feedback. Bioniko (https://www.bioniko.com/, accessed as of June 8, 2023), Phillips Studio (https://phillipsstudio.co.uk/, accessed as of June 8, 2023), and other devices are famous eyeball models constructed from marketed products, some of which were used in several past reports [9, 10]. These models were high quality but relatively expensive [11], while spherical silicone molds were likely to be available on various online markets for approximately $2–10 at the time of this experiment (Table 3). Our results showed that nonvitreoretinal experts mainly value the cost-effectiveness, and together, these facts suggest that anyone can casually purchase silicone molds in many countries. Rice et al. also reported a similar low-cost simulator with an open-sky structure [11]. However, this eye model was crafted from a table tennis ball and had a 40 mm diameter, which seemed to differ in terms of size and rigidity when compared to an actual eyeball.

**Table 3:** Price list of spherical silicone molds in typical online shops

Homepage address	Price
https://item.rakuten.co.jp/mitsuki-nail/20190226001/	229 yen
https://www.pixiecrafting.com/silicone-mold-sphere-25 mm	2,45 €
https://www.amazon.com/dp/B08VGHLJKL?tag=picclick0f-20&linkCode=osi&th=1	$7.99

These were accessed as of February 14, 2022.

This report has some obvious limitations. The simple shape of the model, which does not take much time and effort to construct, may introduce further creative uses, but its form lacks the cornea, lens, and vitreous body. Additionally, the evaluation of the viscosity of the membrane or the rigidity of the pseudo-eyeball wall is not scientific but subjective because we do not have a device to measure these aspects. Moreover, due to the small number of evaluators and lack of comparisons, further investigation in many other facilities is needed. However, this report reveals the simplicity and cost-effectiveness of our simulator, and the results of this questionnaire substantiated its practicality. Our custom-made simulator seemed to have the potential to create an ideal training environment without necessitating travel to special facilities that offer plenty of pig eyes and vitreous surgical machines. We hope that this report will help young vitreous surgeons and patients worldwide.

## Electronic supplementary material

Below is the link to the electronic supplementary material.

Supplementary Material 1

## Data Availability

The datasets generated and/or analysed during the current study are not publicly available but are available from the corresponding author on reasonable request.
